# HIV and Tuberculosis Coinfection in Brazil: Cohort Profile Monitored by the Unified Health System (2015–2018)

**DOI:** 10.1007/s10900-026-01585-8

**Published:** 2026-05-24

**Authors:** Evelyn Lima de Souza, Maria Ines Battistella Nemes, Ana Maroso Alves, Ana Paula Sayuri Sato

**Affiliations:** 1 School of Public Health, Graduate Program in Public Health, University of São Paulo, Av. Dr. Arnaldo, 715, Cerqueira César, São Paulo São Paulo, Brazil; 2 Department of Preventive Medicine, School of Medicine, University of São Paulo, São Paulo, São Paulo, Brazil; 3 Department of Epidemiology, School of Public Health, University of São Paulo, São Paulo, São Paulo, Brazil

**Keywords:** Tuberculosis, HIV, Coinfection, Unified health system, Health information systems

## Abstract

To describe the sociodemographic and clinical profile of individuals with HIV-tuberculosis coinfection followed in services of the Brazilian Unified Health System (SUS), through the integration of data from the Qualiaids-Brazil Cohort and national health information systems. Methods: Nationwide observational study using data from the Qualiaids-BR Cohort, composed of people living with HIV/AIDS (PLWHA) who initiated antiretroviral therapy (ART) between 2015 and 2018 in SUS services participating in the Qualiaids 2016/2017 survey. Individuals with at least one episode of active tuberculosis after ART initiation were included. Results: A total of 7,747 individuals with HIV-tuberculosis coinfection were identified. The majority were men, young, mixed-race, with low educational levels, and residing in the Southeast region. Social vulnerabilities included alcohol, tobacco, or illicit drug use (> 20%). Half presented an initial viral load > 100,000 copies/mL and 60% had baseline CD4 counts < 200 cells/mm³. Regarding tuberculosis, 80.3% were new cases, 9.5% re-entries, and 5.7% relapses. Treatment duration was concentrated between 6 and 12 months, with a cure rate of 70.9% and abandonment in 10.4% of cases, of which 21.3% also discontinued ART. Conclusion: Data integration enabled a comprehensive profile of HIV-tuberculosis coinfection, consistent with national findings and marked by social vulnerabilities that impact adherence and outcomes. The high proportion of treatment abandonment, also associated with ART discontinuation, highlights systemic weaknesses and the need for integrated care strategies for individuals with HIV-tuberculosis coinfection. The findings reinforce the importance of promoting comprehensive and continuous care to mitigate the impacts of coinfection.

## Introduction

Tuberculosis remains one of the leading causes of morbidity and mortality among people living with HIV/AIDS (PLWHA), with an estimated risk 16 times higher compared to the general population [1]. Although preventable and curable, with accessible diagnostic tools and well-established therapy, tuberculosis continues to pose a global challenge, particularly in low- and middle-income countries. The World Health Organization (WHO) highlights it as a global priority, especially in regions with high HIV prevalence. It is estimated that, in 2023, 10.8 million people developed tuberculosis worldwide, resulting in 1.25 million deaths, including 161,000 among PLWHA [1].

Brazil is among the 30 countries with the highest tuberculosis and HIV-tuberculosis burden in absolute numbers, with an average of 8,000 new cases of coinfection annually between 2010 and 2020, of which fewer than 65% were receiving antiretroviral therapy (ART) during this period [2, 3]. The prevalence of HIV-tuberculosis coinfection is higher among men, non-white individuals, those with lower levels of education and income, young adults, and heterosexuals [2, 4–6].

Historic advances in tuberculosis control in Brazil, including BCG (Bacillus Calmette-Guérin) vaccine, its inclusion in the list of mandatory notifiable diseases, and the development of standardized therapeutic regimens, have been crucial in reducing disease transmission and progression [7, 8]. The HIV/AIDS epidemic and the recognition of tuberculosis as a global emergency by the WHO in the early 90’s prompted the creation of specific guidelines for coinfection as part of the implementation of the National Program on Sexually Transmitted Diseases and AIDS of the Ministry of Health. This program focused on mobilizing prevention, treatment, and human rights protection actions for PLWHA, as well as the universal and free introduction of ART [7–10].

Robust information systems with continuous recording have been implemented, enabling epidemiological and clinical monitoring of PLWHA and individuals undergoing tuberculosis treatment in Brazil. The main information systems are [11]: the Notifiable Diseases Information System (SINAN), which records the identification of cases of diseases and conditions included in the national list of mandatory notifiable diseases; the Mortality Information System (SIM), which records deaths; the Medication Logistics Control System (SICLOM), which registers the dispensing of ART medications to all individuals in the country; and the Laboratory Test Control System (SISCEL), which records the results of viral load (VL) and CD4 T-lymphocyte tests of individuals monitored by services within the Unified Health System (SUS).

The nationwide scope, the abundance of relevant data, and the continuous nature of these systems are recognized as strengths of SUS [2, 12]. Analyses based on the integration of data from different systems allow for enhanced epidemiological surveillance and healthcare delivery. However, there is still limited integration between HIV/AIDS and tuberculosis data [5, 6, 13].

In order to contribute to addressing this gap, the present study aims to describe the sociodemographic and clinical profile of individuals with HIV-tuberculosis coinfection treated in SUS services, through the integration of data from the Qualiaids-Brazil Cohort and national health information systems.

### Methods

This is an observational, nationwide study that used data from the Qualiaids-Brazil Cohort (Qualiaids-BR Cohort): an open cohort of PLWHA who initiated ART between 2015 and 2018 and underwent clinical and laboratory follow-up in SUS services that responded to the national Qualiaids survey in 2016/2017 [14–17].

For the construction of the Qualiaids-BR Cohort population, the linked and anonymized data from SINAN (HIV and tuberculosis), SICLOM, SISCEL, and SIM, as well as the separate databases of SICLOM and SISCEL for PLWHA, were provided to the research team by the technical staff of the Department of HIV/AIDS, Tuberculosis, Viral Hepatitis, and Sexually Transmitted Infections (DATHI) of the Ministry of Health.

From the raw linked database, the research team conducted a consistency analysis of the data by cross-referencing information from SICLOM and SISCEL, aiming to identify errors in probabilistic linkage and in the original records of the information systems, thereby qualifying the data for the description of the national cohort of PLWHA. After excluding inconsistencies, the healthcare system responsible for patient follow-up (SUS or private) was defined [16], selecting those monitored within SUS. Finally, individual data were deterministically linked to the primary data from services that responded to the Qualiaids 2016/2017 survey, through the address of the reference service present in both sources, thus selecting individuals whose follow-up was conducted by Qualiaids respondent services [14, 16].

For the selection of the population with HIV-tuberculosis coinfection, the following individuals were excluded from this study:

I. Those without a SINAN record of at least one episode of active tuberculosis infection during the study period, regardless of whether it had already begun before or after HIV diagnosis and/or initiation of ART dispensing, if treatment completion occurred after ART initiation;
II. Those whose tuberculosis treatment completion was recorded as “change of diagnosis”;
III. Those with inconsistencies in tuberculosis data, such as errors in date records and/or in the reason for treatment completion.

**Fig. 1:**
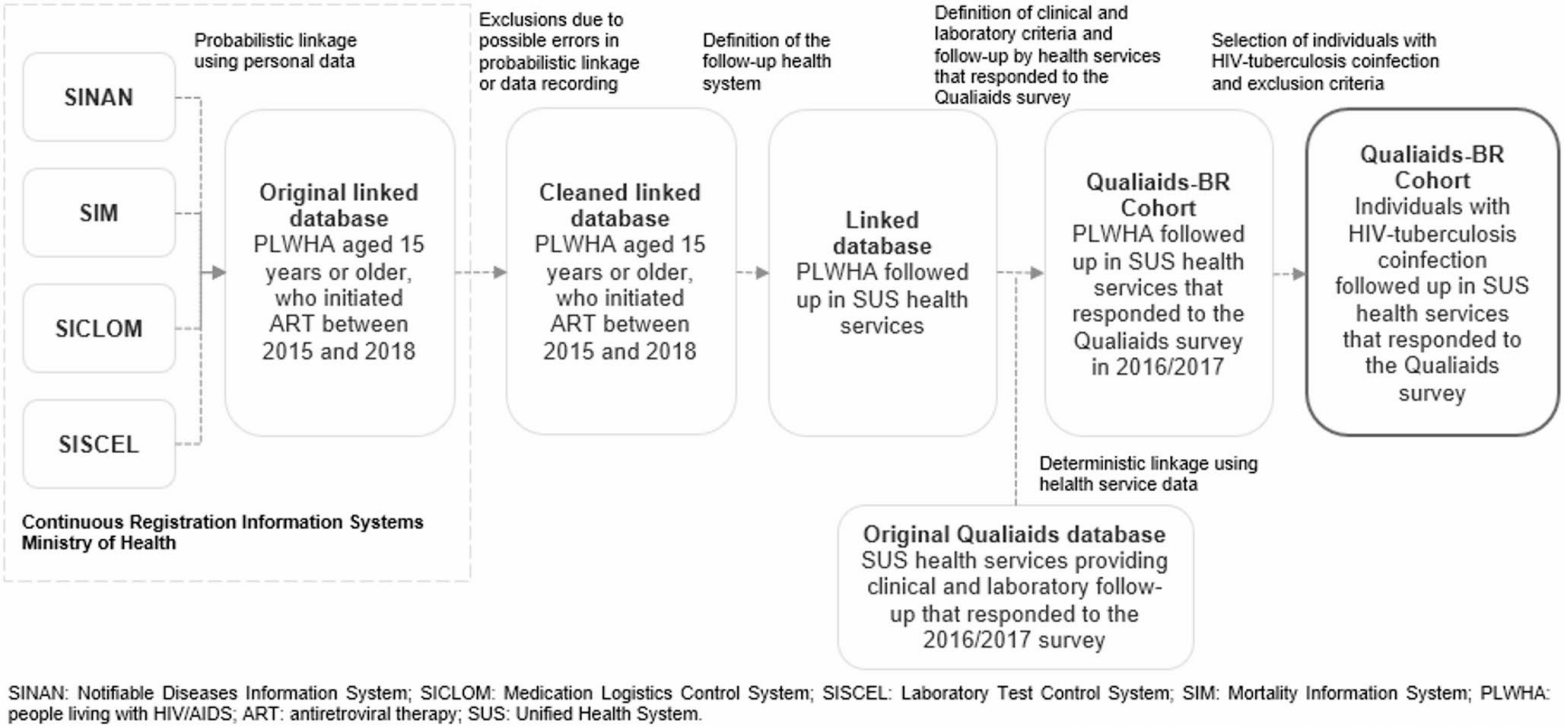
Data sources linkage process, Qualiaids-BR Cohort, 2022

Figure 1 illustrates the process of linking data sources to obtain the Qualiaids-BR Cohort population by the research team, as well as the selection of the population with HIV-tuberculosis coinfection that composes the present study.

Table 1 presents the variables included in the study.

**Table 1:** Variables included for the description of the population profile

Variable	Description	Categories
Sex	Sex	Male; Female; No information
Age	Age in years, grouped into ranges	15–19; 20–29; 30–39; 40–49; 50–59; 60 or more
Race/skin color	Self-reported race/skin color	White; Black; Mixed-race; Yellow; Indigenous; No information
Education	Years of schooling	None; 1–3; 4–7; 8–11; 12 or more; No information
Region of residence	Geographic macro-region of residence	North; Northeast; Midwest; Southeast; South
Deprived of liberty	Person deprived of liberty	No; Yes; No information
Homeless	Person living on the streets	No; Yes; No information
Immigrant	Immigrant status	No; Yes; No information
Alcohol abuse	Alcohol abuse	No; Yes; No information
Tobacco use	Smoker	No; Yes; No information
Mental illness	Person with mental disorder	No; Yes; No information
Diabetes	Person with diabetes	No; Yes; No information
Illicit drug use	Use of illicit drugs	No; Yes; No information
Initial CD4 count	Result of first CD4 test	Less than 200 cells/mm³; 200 to 349 cells/mm³; 350 to 499 cells/mm³; Greater than 499 cells/mm³
Initial viral load	Result of first viral load test	≤ 100,000 copies/mL; >100,000 copies/mL; No information
Definitive ART abandonment	Whether the patient definitively abandoned ART	No; Yes
Reason for treatment entry	Reason for starting tuberculosis treatment	New case; Re-entry after abandonment; Relapse; Transfer; Unknown; Death
Previous tuberculosis episodes	Record of previous tuberculosis episodes during cohort	No; Yes
Tuberculosis treatment duration	In months, calculated by start and end dates	≤ 6; 7–12; 13–18; ≥19; Not possible to calculate
Tuberculosis clinical form	Clinical form of tuberculosis	Pulmonary; Extrapulmonary; Pulmonary + Extrapulmonary
Directly observed therapy (DOT)	DOT performed	No; Yes; No information
Drug sensitivity/resistance test	Drug sensitivity/resistance testing	Resistant to Isoniazid only; Resistant to Rifampicin only; Resistant to both; Resistant to other first-line drugs; Sensitive; Ongoing; Not performed; No information
Reason for tuberculosis treatment completion	Reason for treatment outcome	Cure; Abandonment; Primary abandonment; Death from tuberculosis; Death from other causes; Transfer; Regimen change; Drug-resistant tuberculosis; Treatment failure; No information
Type of HIV/AIDS follow-up health unit	Type of health service where HIV/AIDS follow-up was conducted	Specialized outpatient clinic for HIV/AIDS, STDs and Viral Hepatitis (SAE); Specialized outpatient clinic for infectious diseases; Specialized outpatient clinic for HIV/AIDS, STDs and Viral Hepatitis within a primary care service; Primary care service following HIV/AIDS patients; Outpatient clinic with multiple specialties; Hospital outpatient clinic; Other
HIV/AIDS and tuberculosis treatment in the same service	Whether HIV/AIDS and tuberculosis are treated in the same health service	Treated in the same service by the same physician; Treated in the same service by different physicians; Not treated in the same service

In cases of individuals with more than one episode of tuberculosis during the study period, the most recent record was considered for analysis, representing the most current health condition and treatment status. The variable “previous episodes of tuberculosis” was created considering the occurrence of one or more episodes of tuberculosis after entry into the cohort. Thus, while relapse and re-entry after treatment abandonment (recorded in SINAN) refer to individuals previously treated at any point in life [18], the new variable encompasses only episodes identified after the initiation of ART between 2015 and 2018.

The variables of baseline CD4 count and initial VL were included regardless of the time range between the first and last test, as well as the test result at the time of tuberculosis treatment completion, acknowledging also that the number of tests performed per individual may vary.

The definitions of the reasons for initiation and completion of tuberculosis treatment followed the Ministry of Health’s Manual of Recommendations for Tuberculosis Control [18].

The profile was described using absolute and relative frequencies, mean, standard deviation, median, and range. Analyses were performed with RStudio Desktop version 2024.4.2.764 [19].

The project was approved by the Research Ethics Committee on Human Subjects of the School of Medicine, University of São Paulo, on January 23, 2020 (CAAE: 27659220.3.0000.0065; Approval: 3.807.435).

### Results

**Fig. 2:**
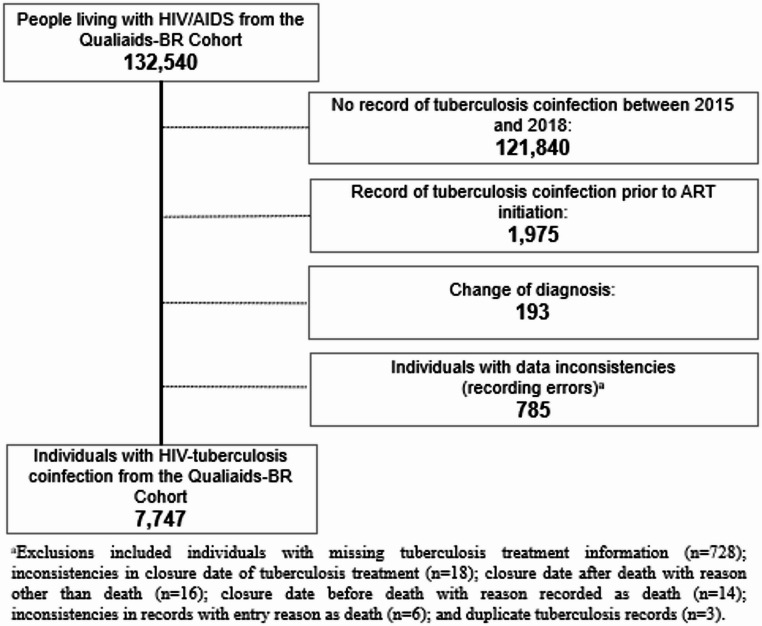
Study population selection, Qualiaids-BR Cohort, 2022

The population with HIV-tuberculosis coinfection comprised 7,747 individuals, representing 5.8% of the total PLWHA included in the Qualiaids-BR Cohort (*n* = 132,540) (Fig. 2).

The sociodemographic profile (Table 2) indicated a predominance of males, aged 20 to 49 years, of mixed race/color, and with 4 to 7 years of schooling. Regarding geographic distribution, most lived in the Southeast region, with more than half from the state of São Paulo.

**Table 2:** Characteristics of individuals with HIV-tuberculosis coinfection in the Qualiaids-BR Cohort, 2015–2018

Characteristics	*N* (7,747)	%
*Sex*		
Male	6,002	77.5
Female	1,744	22.5
No information	1	0.0
*Age (years)* ^a^		
15 to 19	127	1.7
20 to 29	1,883	24.3
30 to 39	2,711	35.0
40 to 49	1,846	23.8
50 to 59	886	11.4
60 or more	294	3.8
*Race/skin color*		
White	2,397	30.9
Black	1,020	13.2
Yellow	52	0.7
Mixed-race	3,696	47.7
Indigenous	12	0.1
No information	570	7.4
*Education (years of schooling)*		
None	151	1.9
1 to 3	826	10.7
4 to 7	2,303	29.7
8 to 11	2,023	26.1
12 or more	693	9.0
No information	1,751	22.6
*Region of residence*		
North	1,160	15.0
Northeast	1,730	22.3
Midwest	361	4.7
Southeast	2,892	37.3
South	1,604	20.7
*Vulnerable populations* ^b^		
Deprived of liberty	324	4.2
Homeless	391	5.0
Immigrant	54	0.7
*Associated conditions* ^c^		
Alcohol abuse	1,843	23.8
Tocacco use	1,916	24.7
Mental illness	246	3.2
Diabetes	193	2.5
Illicit drug use	1,593	20.6
*Initial CD4 count*		
Less than 200 cells/mm³	4,623	59.7
200 to 349 cells/mm³	1,448	18.7
350 to 499 cells/mm³	818	10.6
Greater than 499 cells/mm³	858	11.0
*Initial viral load*		
≤ 100,000 copies/mL	3,837	49.5
> 100,000 copies/mL	3,910	50.5
*Definitive ART abandonment*		
No	6,970	90.0
Yes	777	10.0
*Reason for treatment entry*		
New case	6,221	80.3
Re-entry after abandonment	736	9.5
Relapse	438	5.7
Transfer	327	4.2
Unknown	17	0.2
Death	8	0.1
*Previous tuberculosis episodes*		
No	7,521	97.1
Yes	226	2.9
*Tuberculosis treatment duration (months)*		
≤ 6	4,560	58.9
7 to 12	2,491	32.1
13 to 18	235	3.0
19 or more	38	0.5
No information^d^	423	5.5
*Tuberculosis clinical form*		
Pulmonary	5,078	65.5
Extrapulmonary	1,774	22.9
Pulmonary + Extrapulmonary	895	11.6
*Directly observed therapy*		
No	3,013	38.9
Yes	2,004	25.9
No information	2,730	35.2
*Drug sensitivity/resistance test*		
Resistant to Isoniazid only	32	0.4
Resistant to Rifampicin only	18	0.2
Resistant to both	31	0.4
Resistant to other first-line drugs	36	0.5
Sensitive	910	11.7
Ongoing	98	1.3
Not performed	2,477	32.0
No information	4,145	53.5
*Reason for tuberculosis treatment completion*		
Cure	5,494	70.9
Abandonment	777	10.0
Primary abandonment	29	0.4
Death from tuberculosis	47	0.6
Death from other causes	434	5.6
Transfer	497	6.4
Regimen change	116	1.5
Drug-resistant tuberculosis	91	1.2
Treatment failure	5	0.1
No information	257	3.3
*Type of HIV/AIDS follow-up health unit*		
Specialized outpatient clinic for HIV/AIDS, STDs and Viral Hepatitis (SAE)	3,800	49.0
Specialized outpatient clinic for infectious diseases	1,074	13.9
Specialized outpatient clinic for HIV/AIDS, STDs and Viral Hepatitis within a primary care service	370	4.8
Primary care service following HIV/AIDS patients	314	4.0
Outpatient clinic with multiple specialties	591	7.6
Hospital outpatient clinic	926	12.0
Other	672	8.7
*HIV/AIDS and tuberculosis treatment in the same service*		
Treated in the same service by the same physician	4,280	55.2
Treated in the same service by different physicians	1,820	23.5
Not treated in the same service	1,647	21.3

^a^Mean age of 38 years (standard deviation: 11 years) and range from 15 to 83 years.

^b^Percentage without information: 4.9%, 5.1%, and 6.4%, respectively.

^c^Percentage without information: 5.6%, 6.4%, 5.0%, 4.9%, and 6.8%, respectively.

^d^Absence of treatment start and/or end date, making calculation impossible.

Among populations in situations of vulnerability to tuberculosis, the prevalence of people experiencing homelessness, those deprived of liberty, and immigrants did not exceed 5%. Among conditions associated with coinfection, more than 20% of the population had records of smoking, alcoholism, and illicit drug use.

Regarding HIV/AIDS clinical conditions, most individuals had an initial viral load greater than 100,000 copies/mL and a baseline CD4 T-cell count below 200 cells/mm³. For 10% of PLWHA, there was definitive discontinuation of ART.

With respect to tuberculosis clinical conditions, 80.3% were new cases, 9.5% re-entered after treatment abandonment, and 5.7% were relapsed. Approximately 3% of individuals presented more than one prior episode of active tuberculosis during the cohort period, ranging from 1 episode (*n* = 215 individuals) to 2 episodes (*n* = 11).

The duration of tuberculosis treatment ranged from less than one month (5.7%) to 40 months (one case). More than half completed treatment within six months, while one-third required 7 to 12 months, indicating that nearly all individuals with HIV-tuberculosis coinfection completed tuberculosis treatment within one year.

Pulmonary tuberculosis was identified as the most frequent form, and among these cases, 61.7% completed treatment within six months. The second most frequent clinical form was extrapulmonary tuberculosis, with 53.3% completing treatment within six months, and peripheral lymph node tuberculosis being the most common manifestation (33.8% of extrapulmonary cases).

Approximately one-third of the population did not undergo drug sensitivity/resistance testing, while the same proportion did not receive directly observed therapy (DOT). The proportion of missing information for these variables was 53.5% and 35.2%, respectively.

Treatment outcomes indicated just over 70% cure, while treatment abandonment accounted for about 10% of cases. Among individuals who abandoned treatment (*n* = 806), 21.3% also discontinued ART, 64.0% had initiated tuberculosis treatment as new cases, and 24.8% had initiated treatment as re-entry after abandonment.

The median duration of tuberculosis treatment among cases closed as cure was 205 days (approximately 7 months), while those closed as abandonment and primary abandonment had medians of 151 days (5 months) and 30 days, respectively. Cases closed as transfer had a median of 118 days of treatment (about 4 months), whereas deaths due to tuberculosis, deaths from other causes, and drug-resistant tuberculosis cases presented median treatment durations of 62, 72, and 83 days, respectively (less than 3 months).

Approximately 3% of cases did not have a recorded reason for treatment completion. Of these, 208 (80.9%) lacked a record of the completion date at the time the data were provided; however, 180 (70.0%) had initiated treatment between 2015 and 2017, which could indicate that these individuals had already completed treatment, since most cases are completed within 12 months.

Regarding the chronology of diagnosis, 66.2% of individuals were diagnosed with tuberculosis after the HIV diagnosis date, with 29.1% having less than one month between diagnoses. In addition, 5.1% had both diagnoses recorded on the same date.

Almost half of the individuals were followed for HIV/AIDS in outpatient services exclusively dedicated to specialized care for patients with HIV/AIDS, STIs, and Viral Hepatitis (SAE).

More than half were followed in services that reported managing coinfection treatment within the same service, assisted by the same physician for both conditions. Nearly one-fourth were followed in services that reported managing coinfection treatment within the same service but assisted by different physicians (one for HIV/AIDS and another for tuberculosis). Another 21% were followed for HIV/AIDS in services that reported not managing coinfection treatment within their own facilities.

## Discussion

The availability and unprecedented linkage of secondary data on individual characteristics with primary service data obtained through Qualiaids allowed the profiling of the Qualiaids-BR Cohort of individuals with HIV-tuberculosis coinfection followed in SUS services.

The sociodemographic profile of this population is consistent with national findings [2]. The high prevalence of conditions such as alcoholism, smoking, and illicit drug use reinforces the relationship between social vulnerabilities that favor not only the occurrence of tuberculosis but also a higher risk of unfavorable outcomes, such as treatment abandonment and death [20–22]. Regarding other associated conditions, the small proportion of cases with diabetes and mental illness may be attributed to greater difficulty in collecting this information through self-report or established diagnosis at the time of notification. These conditions have been described as factors associated with a lower likelihood of treatment abandonment, possibly due to more consistent contact with health services for the management of these conditions [13, 23, 24].

More than 60% of individuals had a recorded tuberculosis diagnosis date later than the HIV diagnosis date, and for one-third of these cases the interval was less than one month, indicating the temporal proximity with which individuals are identified with tuberculosis coinfection after the discovery of HIV seropositivity. National data from 2015 to 2018 show that just under half of HIV-tuberculosis coinfection cases had their HIV diagnosis as a result of the tuberculosis diagnosis, reflecting barriers to timely access to diagnosis and treatment [2]. A national study by Saraceni et al. [5] identified that 57.5% of individuals with coinfection between 2011 and 2014 were diagnosed with HIV before tuberculosis, and 38.2% had concomitant diagnoses. In this respect, within the Qualiaids-BR Cohort, the greater identification of tuberculosis cases after HIV diagnosis may be attributed to the study design itself.

Although new cases are more frequent, the proportions of re-entry after abandonment and relapses suggest challenges regarding adherence and therapeutic success, demanding effective strategies for monitoring and tuberculosis prevention among PLWHA [21, 23, 25]. The monitoring of preventive tuberculosis treatment in PLWHA with CD4 ≤ 350 cells/mm³, implemented by the Ministry of Health through the Clinical Monitoring System for people living with HIV/AIDS (SIMC) since 2020, emerges as a promising strategy to reduce the incidence and recurrence of tuberculosis in individuals with latent infection [2].

Regarding tuberculosis treatment duration, most cases were completed between six and twelve months, as established by national protocols, although treatment time may be extended in cases of extrapulmonary forms, drug-resistant tuberculosis regimens, or unsatisfactory clinical progression [18, 26]. The median treatment duration shows important differences depending on the outcome achieved. In cases closed as cure, the median confirms the period required for treatment consolidation and therapeutic success. In contrast, outcomes related to abandonment highlight specific challenges: while abandonment suggests a loss to follow-up occurring even after a considerable treatment period, primary abandonment indicates an early discontinuation, possibly associated with initial difficulties in adherence and linkage to care. Cases of transfer and death also tend to occur earlier, suggesting the need for immediate interventions to identify and mitigate critical factors that compromise treatment continuity and life protection.

Pulmonary tuberculosis was the most frequent form among cases, followed by extrapulmonary tuberculosis, which is consistent with national data. It is also known that individuals with coinfection present a higher proportion of the extrapulmonary clinical form compared to those with tuberculosis without HIV coinfection, which often requires a longer treatment duration to achieve a more satisfactory clinical response [2].

Drug sensitivity/resistance testing is a crucial tool for tuberculosis management, especially in vulnerable populations such as individuals with HIV-tuberculosis coinfection. Identifying the drug sensitivity profile allows the configuration of therapeutic regimens, tailoring treatment to the specific characteristics of each patient. However, the data reveals that one-third of cases did not undergo testing, and in just over half of the records the testing status remains unknown. This gap not only hinders the adoption of individualized therapeutic strategies but also highlights weaknesses in data collection and documentation. Thus, it reinforces the need to train health teams and standardize recording protocols, to ensure proper evaluation of all patients and, consequently, strengthen the control of adverse outcomes, particularly in the context of drug-resistant tuberculosis.

It was evidenced that one-third of the population did not undergo DOT, which is indicated for cases with low adherence to the therapeutic regimen. DOT has proven to be a strategy for strengthening the bond with health services, reducing the risk of treatment abandonment [2, 13, 21, 22, 24, 27].

Regarding tuberculosis treatment outcomes, as expected, most cases achieved cure, while those that evolved into abandonment highlight the need for more effective strategies to ensure adherence and treatment continuity [18, 26]. Among individuals who abandoned treatment, more than 20% also discontinued ART during the cohort, suggesting that systemic factors may simultaneously impact adherence to both therapies in these cases - for example, social determinants reflecting greater socioeconomic vulnerability, fragile self-perception of health and lack of access to services, as well as probable factors related to how care is provided in HIV/AIDS treatment services that weaken patient engagement and generate dissatisfaction with treatment [28]. Monitoring adherence and implementing strategies that consider the uniqueness of individuals in treatment, in addition to approaches that promote shared and comprehensive care between HIV/AIDS and tuberculosis programs, are essential to mitigate the negative impacts of care discontinuity across different contexts.

The absence of information regarding the reason for treatment completion, although proportionally minor in the study population (about 3% of cases), indicates the need to improve the completeness and timeliness of outcome records, especially considering that most of these cases began treatment at the start of the cohort and remained without this information until the data were obtained.

Regarding the typology of treatment services, almost half of the individuals were followed in outpatient clinics exclusively dedicated to specialized care, reflecting the legacy of HIV/AIDS programs, in which most services in the country are focused on this condition, operating as specialized facilities, with few services located at the primary care level.

According to the Qualiaids survey, more than half of the individuals were receiving HIV and tuberculosis treatment within the same service, followed by the same physician for both conditions, which generally favors continuity of care and better guidance when provided by the same team. On the other hand, a significant portion of individuals were followed for HIV/AIDS in services that reported not treating coinfection. In the care of people with HIV-tuberculosis coinfection, fragmentation of assistance is a matter of concern for achieving therapeutic goals, as lack of integration among professionals and unsatisfactory provision of resources for comprehensive and resolutive care are common [28].

The study presents limitations arising from inconsistencies or missing data in routine surveillance records, already noted by previous studies [29–32]. The cohort construction methodology minimized these issues, since for some data more than one source was available for verification, such as registration data or loss to follow-up in SINAN and SICLOM. Nevertheless, relevant frequencies of missing data remained. Additionally, the relatively recent incorporation of fields into the SINAN registration form at the time the Cohort began - such as certain categories of vulnerable populations, associated diseases and conditions, drug sensitivity testing, reasons for treatment completion (regimen change, treatment failure, and primary abandonment), and DOT implementation [33] - may have exacerbated the situation. The intrinsic limitations of the Qualiaids-BR Cohort design have already been discussed in previous publications [14–17].

Despite the limitations, the unprecedented linkage among the robust SUS databases allowed for a comprehensive profile of individuals with HIV-tuberculosis coinfection connected to public health services. The profile highlights gaps in care and underscores the importance of strengthening services by ensuring comprehensive care that prioritizes quality, access, and continuous follow-up. In particular, specialized HIV services stand out for offering longitudinal care - accompanying individuals throughout their lives - and thus providing a lasting bond that is fundamental for treatment maintenance and the promotion of holistic health. Moreover, the findings of this study reveal significant opportunities for advancing scientific research and improving health practices, guiding the prioritization of managerial and care actions within the Brazilian public health system.

## Data Availability

Data used in this work is not available.
